# The association between hypothyroidism and proteinuria in patients with chronic kidney disease: a cross-sectional study

**DOI:** 10.1038/s41598-022-19226-0

**Published:** 2022-09-02

**Authors:** Natsumi Matsuoka-Uchiyama, Kenji Tsuji, Yizhen Sang, Kensaku Takahashi, Kazuhiko Fukushima, Hidemi Takeuchi, Kenichi Inagaki, Haruhito A. Uchida, Shinji Kitamura, Hitoshi Sugiyama, Jun Wada

**Affiliations:** 1 Department of Nephrology, Rheumatology, Endocrinology and Metabolism, Okayama University Graduate School of Medicine, Dentistry and Pharmaceutical Sciences, 2-5-1 Shikata-cho, Okayama, 700-8558 Japan; 2 Department of Chronic Kidney Disease and Cardiovascular Disease, Okayama University Graduate School of Medicine, Dentistry and Pharmaceutical Sciences, Okayama, Japan; 3 Department of Human Resource Development of Dialysis Therapy for Kidney Disease, Okayama University Graduate School of Medicine, Dentistry and Pharmaceutical Sciences, Okayama, Japan

**Keywords:** Nephrology, Kidney diseases, Endocrine system and metabolic diseases

## Abstract

Hypothyroidism is known to be correlated with kidney function and nephrotic range proteinuria. However, it is uncertain whether non-nephrotic proteinuria is associated with hypothyroidism. This study aimed to evaluate the association of proteinuria and hypothyroidism in chronic kidney disease (CKD) patients. We conducted a cross-sectional study composed of 421 CKD patients in a single hospital with measurements of 24-h urine protein excretion (UP) and thyroid function tests. Spearman correlation analysis revealed that 24-h Cr clearance (24hrCcr) was positively (r = 0.273, *p* < 0.001) and UP was negatively (r = − 0.207, *p* < 0.001) correlated with free triiodothyronine. Frequency distribution analysis stratified by CKD stage and UP for hypothyroidism revealed that the prevalence of hypothyroidism was higher among participants with higher CKD stage and nephrotic range proteinuria. Multivariate logistic regression analysis revealed that 24hrCcr and UP were significantly correlated with hypothyroidism (24hrCcr/10 mL/min decrease: odds ratio [OR], 1.29; 95% confidence interval [CI], 1.18–1.41; UP/1 g increase: OR, 1.10; 95% CI, 1.03–1.17). In addition, nephrotic range proteinuria, but not moderate UP (UP: 1.5–3.49 g/day), was significantly correlated with hypothyroidism compared to UP < 0.5 g/day. In summary, decreased kidney function and nephrotic range proteinuria, not non-nephrotic proteinuria, are independently associated with the hypothyroidism.

## Introduction

It is known that thyroid and kidney function have a strong influence on each other^1^. As the side of the effect of renal function on the thyroid disorder, there are several reports indicating the high prevalence of hypothyroidism in chronic kidney disease (CKD) patients^2–13^. For example, using the data from the Third National Health and Nutrition Examination Survey in US, it is reported that a prevalence of hypothyroidism in persons with eGFR < 60 ml/min/1.73 m^2^ was more than 20% compared to that of 5.4% for persons with eGFR ≥ 90 ml/min/1.73 m^2^ ^9^, indicating a higher prevalence of hypothyroidism in CKD patients. Nevertheless, most of them collected the medical checkup data whose participants were mostly healthy without CKD and there are still few reports analyzing the thyroid function among CKD population^3,6,14^. It is reported that kidney dysfunction may cause hypothyroidism by several mechanisms: high exposure to iodine by the reduction in iodine clearance related to renal dysfunction^15,16^, decrease in thyrotropin-releasing hormone (TRH) by uremia^17,18^, and the reduction in triiodothyronine (T3) concentrations by chronic metabolic acidosis^19^.

In addition to the kidney function, the severity of CKD is also defined by urine protein excretion. It is reported that proteinuria may cause urinary loss of thyroid hormones bound to the various binding proteins such as thyroxine-binding globulin (TBG), albumin, prealbumin, and transthyretin^20^, which result in the reduction of the serum thyroid hormone levels^21^. Nevertheless, there are still few reports analyzing the urine protein and hypothyroidism. While it is reported that a semi-quantitative proteinuria by urine dipstick is not associated with hypothyroidism^4^, the effect of degree of proteinuria on hypothyroidism is still unclear. Although 24-h urinary protein excretion (UP) is desirable for accurate measurement of proteinuria because of fluctuation of urinary protein excretion by measurement time, position, exercise, and blood pressure, it is inconvenient, and spot urinary protein / creatinine ratio is a convenient alternative and is in widespread use^22^. Nevertheless, it is suggested that thyroid function may affect urine creatinine excretion^23^, suggesting that spot urinary protein / creatinine ratio may not reflect accurate proteinuria in the case with hypothyroidism. Therefore, in the present study, we applied UP to analyze the association between urinary protein levels and the presence of hypothyroidism in CKD patients.

## Materials and methods

### Study design and participants

**Figure 1:**
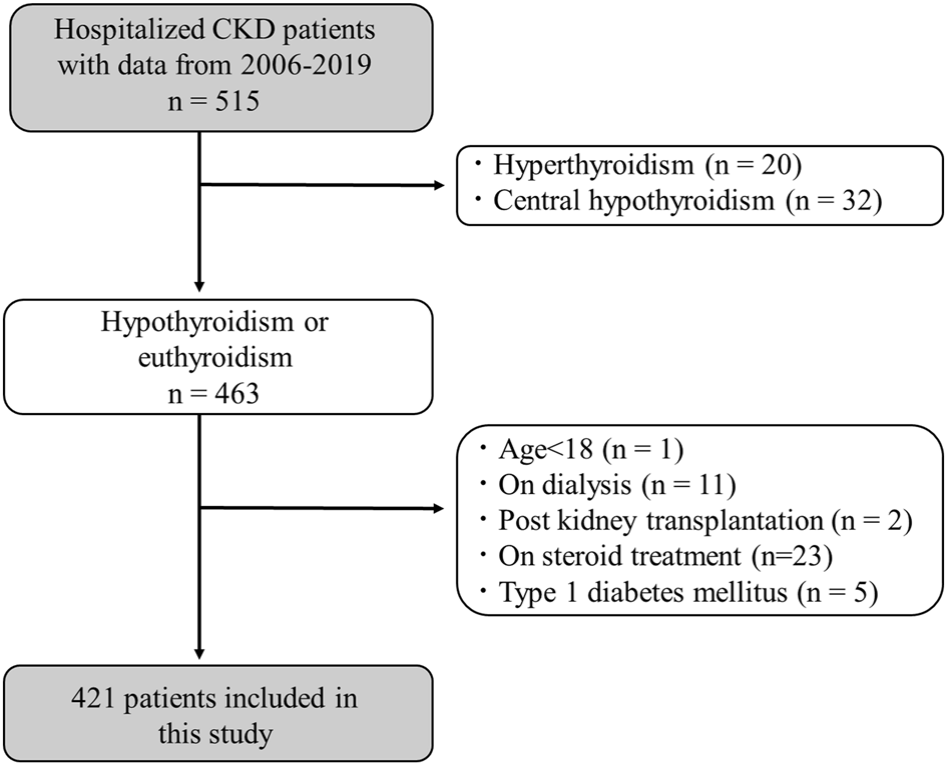
Flow diagram of the screening and enrollment of study patients. CKD, chronic kidney disease.

We retrospectively reviewed patients with measurement of thyroid function and renal function during the hospitalization in the division of kidney, diabetes, and endocrine diseases at Okayama University Hospital from 2006 to 2019. Data were collected from electronic-based records in 2020–2021. Among 18,068 patients from our database, we collected 515 CKD patients with all the following data of the measurements; eGFRcre, eGFRcys, 24hrCcr, thyroid-stimulating hormone (TSH), free thyroxine (FT4), free triiodothyronine (FT3), UP, total cholesterol, serum albumin, and glycated hemoglobin (HbA1c). We extracted 463 hypothyroid and euthyroid patients after excluding patients with hyperthyroidism or central hypothyroidism, followed by the exclusion according to the following criteria: (1) age < 18 years, (2) on dialysis, (3) post kidney transplantation, (4) on steroid treatment, and (5) type 1 diabetes mellitus (DM). Consequently, 421 patients were enrolled in this study (Fig. 1). To analyze the explanatory variables, including UP, as possible exposures for the outcome of the presence of hypothyroidism, univariate and multivariate logistic regression analyses were performed. The protocol of this study was approved by the ethics committees of Okayama University Hospital Institutional Review Board (accredited ISO9001/2000), Okayama, Japan (approval number: OKU-206-022), and no written informed consent was obtained because the study was considered exempt. Instead, the content of the research was posted on our department homepage and the hospital for the public informed consent. This study also followed the Declaration of Helsinki on medical protocol and ethics.

### Data collection

The following clinical characteristics were collected at the time of the hospitalization: age, sex, body mass index (BMI), and the use of angiotensin-converting-enzyme inhibitor (ACE-i), angiotensin II receptor blocker (ARB), insulin, dipeptidyl peptidase-4 (DPP-4) inhibitor, glucagon-like peptide-1 (GLP-1) receptor agonist, biguanide, sodium-glucose cotransporter 2 (SGLT-2) inhibitor, sulfonylurea (SU), thiazolidinedione (TZD), glinide, and thyroid hormone replacement therapy (THRT). HbA1c data are presented as National Glycohemoglobin Standardization Program values according to the recommendations of the Japanese Diabetes Society and International Federation of Clinical Chemistry^24^. The presence of DM was defined as HbA1c ≥ 6.5% and fasting plasma glucose ≥ 126 mg/dL and/or postprandial plasma glucose ≥ 200 mg/dL or prior diagnosis or use of anti-diabetes medications. (In the absence of unequivocal hyperglycemia, diagnosis requires two abnormal test results from the same sample or in two separate test samples). The presence of hypertension was defined with prior diagnosis or use of anti-hypertensive medications. eGFRcre was calculated by using Modified for Japanese subjects: eGFRcre (mL/min/1.73 m^2^) = 194 × s-Cr (mg/dL)^−1.094^ × Age^−0.287^ (× 0.739 for females)^25^. CKD was defined as eGFRcre < 60 mL/min/1.73 m^2^ and/or urinary protein ≥ 0.15 g/day^26^ and CKD stages were defined by a modification of the National Kidney Foundation CKD staging based on estimated GFR categories of ≥ 90, 60 to 89, 45 to 59, 30 to 44, and < 30 mL/min/1.73 m^2^ ^27^. Nephrotic syndrome (NS) was defined by both substantial proteinuria (> 3.5 g/24 h) and hypoalbuminemia (< 3.0 g/dL). eGFRcys was calculated by: eGFRcys (mL/min/1.73 m^2^) = (104 × serum cystatin C (mg/L)^−1.019^ × 0.996 ^age^)—8 (male), (104 × serum cystatin C (mg/L)^−1.019^ × 0.996 ^age^ × 0.929)—8 (female)^28^. 24hrCcr (mL/min) was calculated as follows: 24-h urinary creatinine (mg/dL) × 24-h collected urine (mL/day)/24 (hour)/60 (min)/serum creatinine (mg/dL) × 1.73/body surface area (BSA)^29^. BSA was calculated by: BSA (m^2^) = body weight^0.425^ × height^0.725^ × 0.007184 ^30^. Thyroid function was analyzed by an electrochemiluminescence assay (Roche Diagnostics K.K., Cobas 8000). The normal reference range in our institute for TSH was 0.27–4.2 µIU/mL, FT4 was 0.93–1.7 ng/dL, and FT3 was 2.3–4.0 pg/mL. Participants were classified into 3 groups according to the results of thyroid function test and the use of THRT: subclinical hypothyroidism (0.93 ≤ FT4 ≤ 1.7 ng/dL and TSH > 4.2 µIU/mL), overt hypothyroidism (FT4 < 0.93 ng/dL and TSH > 4.2 µIU/L, and/or on THRT), and euthyroidism (0.93 ≤ FT4 ≤ 1.7 ng/dL and 0.27 ≤ TSH ≤ 4.2 µIU/mL). Total hypothyroidism was defined as combined overt and subclinical hypothyroidism. The etiology for CKD was diagnosed by experienced nephrologists based on patients’ medical history, physical information, clinical examination, and pathological findings by renal biopsy, if applicable.

### Statistical analysis

Data were expressed as n (%) for categorical variables and mean ± standard deviation (SD) for continuous variables. Categorical variables were analyzed with the chi-square test, while continuous variables were compared by using the student's t-test or Mann–Whitney U test as appropriate. P for trend was calculated by Cochran-Armitage trend test or Cuzick test. The estimated standard error of the confidence estimate was used to establish confidence intervals (CI) of the estimated odds ratio (OR). Correlations of renal function and UP with thyroid function (FT4, TSH, FT3) were evaluated by Spearman correlation analysis. The statistical analyses were performed by JMP version 14.0.0 (SAS Institute, Inc, Cary, NC), and Stata/SE version 16.1 (Stata Corp LLC, College Station, TX). All *P* values were calculated as two-sided. The association was considered significant with *P* values less than 0.05.

## Results

### Study Population and clinical characteristics

Among 515 patients with all the data set, 421 patients were included in the eventual analysis after the exclusion. A flow chart of the screening and registration of the study participants is shown in Fig. 1. The characteristics of the participants stratified by CKD stage are shown in Table 1. Underlying diseases in CKD patients are shown in Table S1; diabetic nephropathy (n = 163, 39%), nephrosclerosis (n = 74, 18%), glomerulonephritis (n = 77, 18%), others (n = 44, 10%), and unknown (n = 63, 15%). The number of participants with CKD stage 1, 2, 3, 4, and 5 were 34, 92, 177, 65, and 53, respectively. The average age was 61 ± 15 years and 55% were male. The average eGFRcre was 49.6 ± 30.0 ml/min/1.73 m^2^. The average level of UP was 2.09 ± 3.72 g/day, and 11% subjects were diagnosed with NS. 11% subjects took THRT and the average TSH was 5.6 ± 18.1 µU/mL. The overall prevalence of overt hypothyroidism, subclinical hypothyroidism and total hypothyroidism was 12%, 16% and 28%, respectively. The average HbA1c levels were 6.8% and 48% of subjects had a history of DM.

**Table 1:** Characteristics of the study participants stratified by CKD stages.

Clinical parameters	All CKD (n = 421)	Stage 1 (n = 34)	Stage 2 (n = 92)	Stage 3 (n = 177)	Stage 4 (n = 65)	Stage 5 (n = 53)	*P* value
Sex (Male), n (%)	231 (55)	12 (35)	53 (58)	97 (55)	49 (75)	20 (38)	0.594
Age (yr)	61 ± 15	43 ± 15	55 ± 14	64 ± 14	70 ± 11	63 ± 15	< 0.001**
BMI (kg/m^2^)	24.6 ± 4.9	24.0 ± 4.3	24.5 ± 4.0	24.7 ± 5.0	24.9 ± 6.6	24.5 ± 4.2	0.736
TSH (µU/mL)	5.6 ± 18.1	1.99 ± 1.10	2.33 ± 1.94	6.7 ± 24.0	4.66 ± 7.10	10.18 ± 23.90	< 0.001**
FT4 (ng/dL)	1.18 ± 0.22	1.23 ± 0.17	1.22 ± 0.17	1.18 ± 0.23	1.20 ± 0.24	1.09 ± 0.27	0.008**
FT3 (pg/mL)	2.48 ± 0.62	2.70 ± 0.60	2.67 ± 0.54	2.50 ± 0.65	2.23 ± 0.54	2.21 ± 0.59	< 0.001**
s-Cr (mg/dL)	1.62 ± 1.34	0.55 ± 0.12	0.77 ± 0.13	1.16 ± 0.27	2.25 ± 0.48	4.56 ± 1.37	< 0.001**
eGFRcre (mL/min/1.73 m^2^)	49.6 ± 30.0	113.1 ± 31.7	74.4 ± 8.3	46.1 ± 8.4	23.0 ± 4.6	10.2 ± 2.7	< 0.001**
eGFRcys (mL/min/1.73 m^2^)	49.5 ± 30.4	98.6 ± 21.8	77.1 ± 20.1	47.1 ± 17.2	22.3 ± 8.2	11.6 ± 3.8	< 0.001**
24hrCcr (mL/min)	57.5 ± 36.8	122.1 ± 25.2	87.5 ± 25.6	54.8 ± 21.6	25.7 ± 8.3	12.2 ± 4.4	< 0.001**
Urinary protein (g/day)	2.09 ± 3.72	0.65 ± 1.24	1.66 ± 3.36	1.84 ± 3.62	2.73 ± 8.65	3.86 ± 4.51	< 0.001**
Albumin (g/dL)	3.5 ± 0.8	3.9 ± 0.6	3.7 ± 0.8	3.5 ± 0.9	3.3 ± 0.8	3.5 ± 0.6	< 0.001**
Hemoglobin (g/dL)	12.1 ± 2.4	13.0 ± 2.6	13.2 ± 2.1	12.1 ± 2.2	11.6 ± 2.7	10.8 ± 2.1	< 0.001**
Total cholesterol (mg/dL)	194 ± 62	194 ± 65	208 ± 67	196 ± 64	182 ± 55	177 ± 45	< 0.001**
HbA1c (%)	6.8 ± 1.9	8.3 ± 3.2	7.3 ± 2.0	6.8 ± 1.7	6.3 ± 1.1	5.8 ± 0.7	< 0.001**
Nephrotic syndrome, n (%)	48 (11)	1 (3)	10 (11)	18 (10)	11 (17)	8 (15)	0.054
Diabetes mellitus, n (%)	200 (48)	19 (56)	48 (52)	82 (46)	30 (46)	21 (40)	0.087
Hypertension, n (%)	113 (27)	3 (9)	16 (17)	41 (23)	26 (40)	27 (51)	0.261
ACE-i/ARB intake, n (%)	105 (25)	3 (9)	20 (22)	40 (23)	16 (25)	26 (49)	< 0.001**
**Antidiabetic medication**							
Insulin, n (%)	96 (23)	12 (35)	21 (23)	39 (22)	14 (22)	10 (19)	0.152
DPP-4 inhibitor, n (%)	52 (12)	4 (12)	13 (14)	26 (15)	6 (9)	3 (6)	0.161
GLP-1 receptor agonist, n (%)	5 (1)	1 (3)	2 (2)	1 (1)	1 (2)	0 (0)	0.198
Biguanide, n (%)	33 (8)	5 (15)	8 (9)	19 (11)	0 (0)	1 (2)	0.005**
SGLT-2 inhibitor, n (%)	5 (1)	0 (0)	2 (2)	2 (1)	0 (0)	1 (2)	0.957
SU, n (%)	44 (10)	1 (3)	12 (13)	27 (15)	3 (5)	1 (2)	0.140
TZD, n (%)	13 (3)	0 (0)	3 (3)	7 (4)	3 (5)	0 (0)	0.930
Glinide, n (%)	5 (1)	0 (0)	1 (1)	1 (1)	2 (3)	1 (2)	0.238
**Cause of CKD**							
Diabetic nephropathy, n (%)	163 (39)	19 (56)	41 (45)	65 (37)	24 (37)	14 (26)	0.004**
Nephrosclerosis, n (%)	74 (18)	2 (6)	6 (7)	29 (16)	19 (29)	18 (34)	< 0.001**
Glomerulonephritis, n (%)	77 (18)	7 (21)	28 (30)	30 (17)	4 (6)	8 (15)	0.006**
Others, n (%)	44 (10)	1 (3)	8 (9)	17 (10)	9 (14)	9 (17)	0.021*
Unknown, n (%)	63 (15)	5 (15)	9 (10)	36 (20)	9 (14)	4 (8)	0.649
On THRT, n (%)	45 (11)	0 (0)	4 (4)	18 (10)	9 (14)	14 (26)	< 0.001**
Subclinical hypothyroidism, n (%)	68 (16)	2 (6)	8 (9)	33 (19)	12 (18)	13 (25)	0.003**
Overt hypothyroidism, n (%)	51 (12)	0 (0)	6 (7)	16 (9)	12 (18)	17 (32)	< 0.001**
Total hypothyroidism, n (%)	119 (28)	2 (6)	14 (15)	49 (28)	24 (37)	30 (57)	< 0.001**
Euthyroidism, n (%)	302 (72)	32 (94)	78 (85)	128 (72)	41 (63)	23 (43)	< 0.001**

*CKD* chronic kidney disease, *BMI* body mass index, *TSH* thyroid-stimulating hormone, *FT4* free thyroxine, *FT3* free triiodothyronine, *s-Cr* serum creatinine, *eGFRcre* estimated glomerular filtration rate calculated by serum creatinine, *eGFRcys* estimated glomerular filtration rate calculated by serum cystatin C, *24hrCcr* 24-h creatinine clearance, *HbA1c* glycated hemoglobin, *ACE-i* angiotensin-converting-enzyme inhibitor, *ARB* angiotensin II receptor blocker, *DPP-4* dipeptidyl peptidase-4, *GLP-1* glucagon-like peptide-1, *SGLT-2* sodium-glucose cotransporter 2, *SU* sulfonylurea, *TZD* thiazolidinedione, *THRT* thyroid hormone replacement therapy.

Data was expressed as n (%) for categorical variables and mean ± standard deviation for continuous variable. P for trend was obtained by Cochran-Armitage trend test or Cuzick test. *P < 0.05, **P < 0.01.

### Correlations between kidney function and thyroid disorder

**Figure 2:**
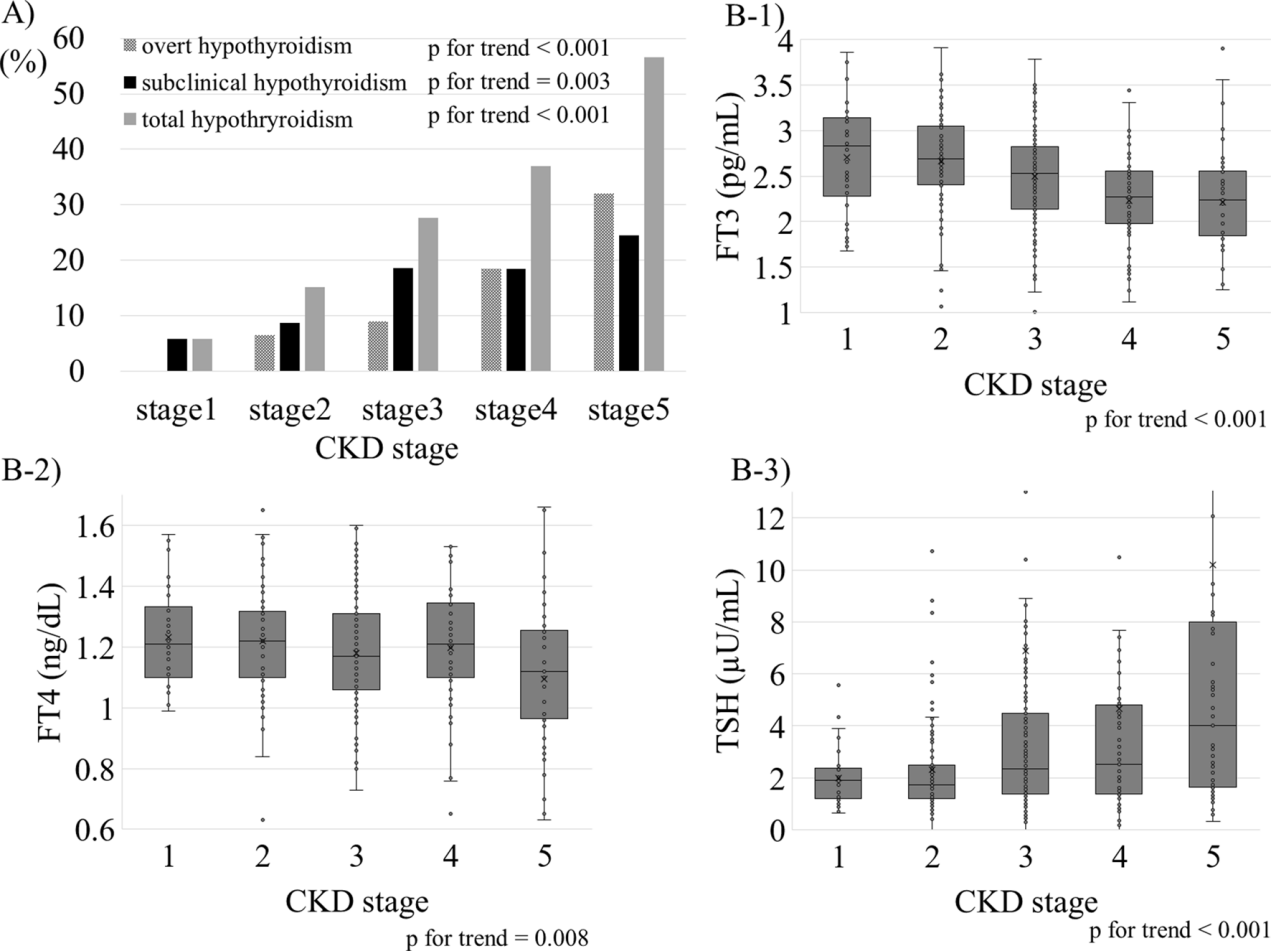
The association between CKD and hypothyroidism. (**A**) Trend of the prevalence of overt hypothyroidism, subclinical hypothyroidism, and total hypothyroidism as per CKD stages. (**B**) Trend test for correlation between FT3, FT4, and TSH as per CKD stages. CKD, chronic kidney disease; TSH, thyroid-stimulating hormone; FT4, free thyroxine; FT3, free triiodothyronine.

**Figure 3:**
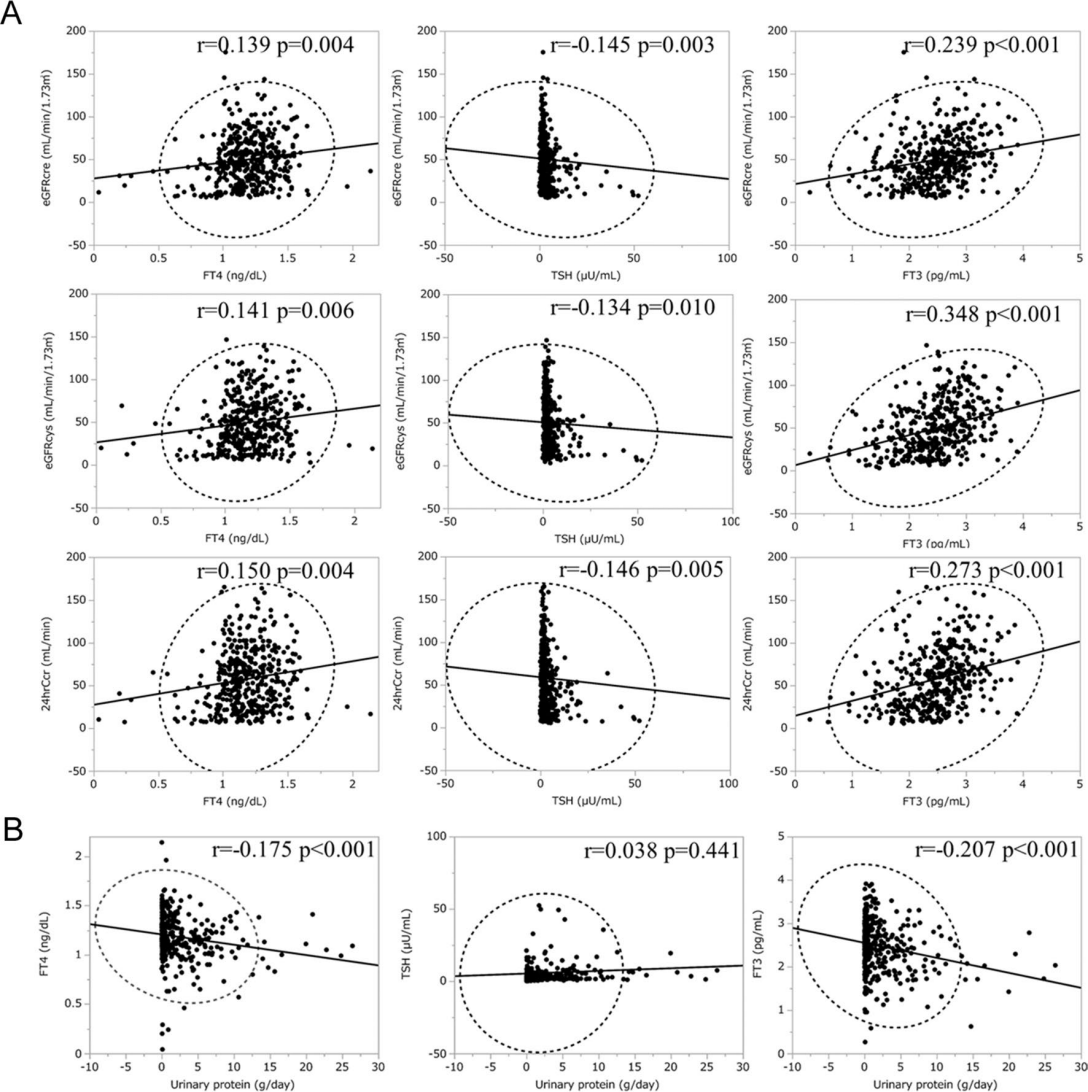
The association between renal function, proteinuria and thyroid function. (**A**) The association between renal function and thyroid function (**B**) The association between proteinuria and thyroid function. eGFRcre, estimated glomerular filtration rate calculated by serum creatinine; eGFRcys, estimated glomerular filtration rate calculated by serum cystatin C; 24hrCcr, 24-h creatinine clearance; TSH, thyroid-stimulating hormone; FT4, free thyroxine; FT3, free triiodothyronine.

We investigated the association between hypothyroidism and CKD. There was a significantly increased proportion of overt, subclinical, and total hypothyroidism along with the higher CKD stage (*P* value for trends < 0.001, 0.003, and < 0.001, respectively) (Fig. 2A), which is compatible with the previous reports^4,9,31^. We then compared TSH and thyroid hormone between the CKD groups. Participants showed lower FT3 and FT4 levels (*P* value for trends < 0.001, and 0.008, respectively) and higher TSH levels (*P* value for trend < 0.001) with higher CKD stage (Fig. 2B). We also conducted scatter plot analysis with regression fit line representing the association between renal function and thyroid function of FT3, FT4 and TSH levels. There were weak correlations between FT3 levels and each renal function measurement; eGFRcre, eGFRcys, and 24hrCcr (r = 0.239; *P* < 0.001, r = 0.348; *P* < 0.001, and r = 0.273; *P* < 0.001, respectively) (Fig. 3), suggesting that FT3 levels are mostly associated with kidney function among these thyroid function tests.

### Logistic regression analysis of hypothyroidism in CKD patients

Next, the explanatory variables for hypothyroidism were evaluated by univariate and multivariate logistic regression analysis (Table 2). In the univariate analysis, the factors associated with the presence of total hypothyroidism were 24hrCCr (OR, 1.30; 95% CI, 1.20–1.41, decreased by 10 mL/min), NS (OR, 2.93; 95% CI, 1.59–5.40), UP (OR, 1.12; 95% CI, 1.06–1.19) and HbA1c (OR, 0.79; 95% CI, 0.69–0.91) while age, sex and BMI were not detected as explanatory variables for total hypothyroidism. NS, UP and 24hrCcr still showed positive correlations with total hypothyroidism even after multivariate adjustment (Table 2). In addition to the 24hrCcr, we also applied different measurements of renal function, eGFRcre and eGFRcys, which revealed that all the measurements were significantly associated with the total hypothyroidism even after the adjustment for variables (Table S2). We also performed a subgroup analysis for subclinical hypothyroidism and overt hypothyroidism in addition to total hypothyroidism (Table S3), indicating positive correlations of UP with both subclinical hypothyroidism and overt hypothyroidism. Furthermore, we also performed a subgroup analysis divided by the presence of DM. Clinical variables stratified the presence of DM and the result of the subgroup analysis are shown in Tables S4 and S5, respectively. While we observed the positive correlations of UP and NS with total hypothyroidism in Non-DM group, there was no correlations in DM group (Table S5).

**Table 2:** Nominal logistic regression model for the presence of total hypothyroidism and variables.

Clinical parameters	OR [95% CI]		
	Univariate	Multivariate Model 1	Multivariate Model 2
Sex (male)	0.90 [0.59–1.37]	0.65 [0.40–1.04]	0.66 [0.41–1.07]
24hrCcr [decreased by 10 mL/min]	1.30 [1.20–1.41]	1.28 [1.17–1.40]	1.29 [1.18–1.41]
Age	1.02 [1.00–1.03]	1.00 [0.99–1.02]	1.00 [0.98–1.02]
BMI	1.01 [0.97–1.05]	1.00 [0.95–1.05]	1.01 [0.96–1.06]
Hypertension	1.98 [1.25–3.13]	1.13 [0.67–1.91]	1.26 [0.75–2.10]
Urinary protein [increased by 1 g/day]	1.12 [1.06–1.19]	1.10 [1.03–1.17]	–
Nephrotic syndrome	2.93 [1.59–5.40]	–	2.49 [1.28–4.84]
HbA1c	0.79 [0.69–0.91]	–	–
Diabetes mellitus	0.77 [0.50–1.18]	0.84 [0.52–1.36]	0.83 [0.52–1.35]

*OR* odds ratio, *95% CI* 95% confidence intervals, *BMI* body mass index, *HbA1c* glycated hemoglobin, *24hrCcr* 24-h creatinine clearance.

Multivariate Model1: Adjusted for sex, 24hrCcr, age, BMI, the presence of hypertension and diabetes mellitus, and urinary protein. Multivariate Model2: Adjusted for sex, 24hrCcr, age, BMI, the presence of hypertension, diabetes mellitus and nephrotic syndrome.

### Correlations between proteinuria and thyroid disorder

**Figure 4:**
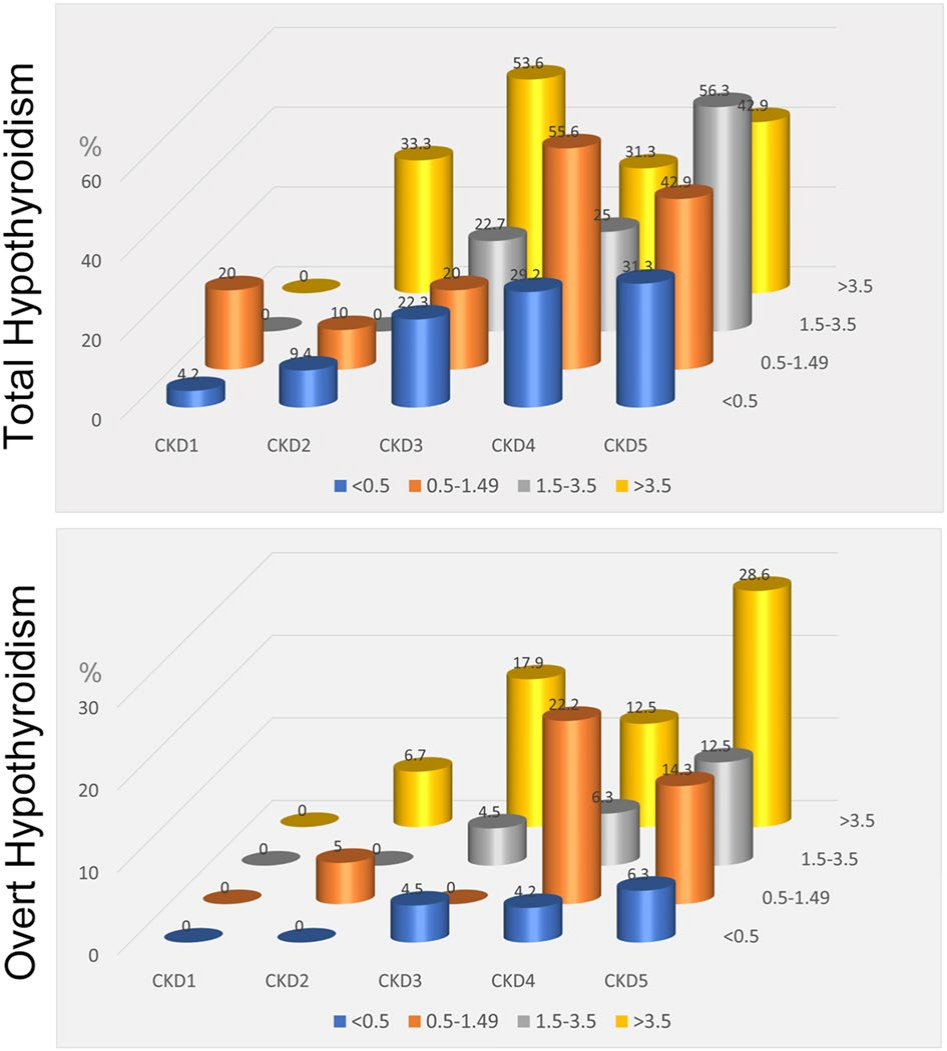
Association of hypothyroidism with CKD stage and urinary protein excretion level. CKD, chronic kidney disease; UP, 24-h urine protein excretion.

To further analyze the association of proteinuria with hypothyroidism, we divided the participants into four categories based on the UP: < 0.5, 0.5–1.49, 1.5–3.49, and 3.5 ≥ g/day. Clinical variables stratified by these categories are shown in Table 3. Etiologies for CKD in patients with NS are shown in Figure S1. Patients with higher UP had higher levels of TSH, s-Cr, total cholesterol; had lower levels of FT4, FT3, eGFRcre, 24hcCcr, albumin, hemoglobin, and HbA1c; had higher prevalence of subclinical and overt hypothyroidism; and took more THRT and ACE-I/ARB. We also conducted scatter plot analysis with regression fit line representing the association between UP and FT3, FT4 and TSH levels. Spearman correlation analysis revealed a weak correlation between FT3 levels and UP (Fig. 3B). Frequency distribution analysis stratified by CKD stage and UP for total and overt hypothyroidism (Fig. 4) revealed the higher prevalence as the higher CKD stage as well as higher UP. Especially, the prevalence of hypothyroidism in the nephrotic range proteinuria category in CKD stage 2–5 was higher than others, suggesting that nephrotic range proteinuria has a strong association with hypothyroidism. Finally, we conducted a logistic regression model for the presence of hypothyroidism and UP divided by 4 categories, indicating that only the nephrotic range proteinuria category had a significant correlation with the presence of total hypothyroidism compared to the reference of the category, UP < 0.5 g/day (Table 4). Taken together, nephrotic range proteinuria, not non-nephrotic proteinuria, is independently associated with the presence of total hypothyroidism. We also performed a subgroup analysis for subclinical hypothyroidism and overt hypothyroidism in addition to total hypothyroidism (Table S6), revealing a positive correlation of nephrotic range proteinuria only with overt hypothyroidism. Furthermore, we also performed a subgroup analysis divided by the presence of DM (Table S7). While we observed the positive correlations of nephrotic range proteinuria with total hypothyroidism in Non-DM group, there was no correlation in DM group.

**Table 3:** Clinical parameters for all patients and for patients stratified by the urine protein levels.

Urine protein (g/day)	All patients (n = 421)	< 0.5 (n = 224)	0.5–1.49 (n = 56)	1.5–3.49 (n = 60)	> 3.5 (n = 81)	*P* value
Sex (Male), n (%)	231 (55)	116 (52)	33 (59)	30 (50)	52 (64)	0.120
Age (yr)	61 ± 15	62 ± 15	57 ± 15	62 ± 17	61 ± 16	0.700
BMI (kg/m^2^)	24.6 ± 4.9	24.3 ± 4.2	23.4 ± 4.1	24.9 ± 5.3	26.1 ± 6.4	0.157
TSH (µU/mL)	5.6 ± 18.1	4.8 ± 17.6	3.5 ± 5.0	9.7 ± 31.9	6.0 ± 8.4	< 0.001**
FT4 (ng/dL)	1.18 ± 0.22	1.21 ± 0.22	1.21 ± 0.26	1.17 ± 0.21	1.10 ± 0.20	< 0.001**
FT3 (pg/mL)	2.48 ± 0.62	2.52 ± 0.63	2.67 ± 0.65	2.41 ± 0.57	2.25 ± 0.54	< 0.001**
s-Cr (mg/dL)	1.62 ± 1.34	1.22 ± 0.77	1.57 ± 1.28	2.15 ± 1.58	2.39 ± 1.89	< 0.001**
eGFRcre (mL/min/1.73 m^2^)	49.6 ± 30.0	56.3 ± 30.4	53.2 ± 28.4	37.6 ± 26.6	37.5 ± 26.2	< 0.001**
eGFRcys (mL/min/1.73 m^2^)	49.5 ± 30.4	55.6 ± 29.3	57.2 ± 34.4	36.9 ± 26.5	36.7 ± 26.1	< 0.001**
24hrCcr (mL/min)	57.5 ± 36.8	65.1 ± 37.6	62.1 ± 36.7	43.4 ± 31.7	43.8 ± 31.5	< 0.001**
Urinary protein (g/day)	2.09 ± 3.72	0.17 ± 0.12	0.92 ± 0.27	2.27 ± 0.59	8.10 ± 4.94	< 0.001**
Albumin (g/dL)	3.5 ± 0.8	3.8 ± 0.6	3.7 ± 0.7	3.4 ± 0.6	2.7 ± 0.9	< 0.001**
Hemoglobin (g/dL)	12.1 ± 2.4	12.5 ± 2.3	12.1 ± 2.3	11.2 ± 2.5	11.9 ± 2.5	0.002**
Total cholesterol (mg/dL)	194 ± 62	183 ± 52	183 ± 46	184 ± 51	239 ± 82	< 0.001**
HbA1c (%)	6.8 ± 1.9	7.3 ± 2.1	6.2 ± 1.2	6.3 ± 1.4	6.4 ± 1.3	< 0.001**
Diabetes mellitus, n (%)	200 (48)	117 (52)	19 (34)	25 (42)	39 (48)	0.019*
Hypertension, n (%)	113 (27)	30 (13)	21 (38)	26 (43)	36 (44)	< 0.001**
ACE-i/ARB intake, n (%)	105 (25)	39 (17)	15 (27)	24 (40)	27 (33)	< 0.001**
**Antidiabetic medication**						
Insulin, n (%)	96 (23)	55 (25)	8 (14)	11 (18)	22 (27)	0.212
DPP-4 inhibitor, n (%)	52 (12)	35 (16)	5 (9)	4 (7)	8 (10)	0.167
GLP-1 receptor agonist, n (%)	5 (1)	2 (1)	1 (2)	1 (2)	1 (1)	0.929
Biguanide, n (%)	33 (8)	28 (13)	2 (4)	0 (0)	3 (4)	0.002**
SGLT-2 inhibitor, n (%)	5 (1)	4 (2)	1 (2)	0 (0)	0 (0)	0.467
SU, n (%)	44 (10)	28 (13)	2 (4)	7 (12)	7 (9)	0.239
TZD, n (%)	13 (3)	8 (4)	0 (0)	4 (7)	1 (1)	0.141
Glinide, n (%)	5 (1)	1 (0)	2 (4)	0 (0)	2 (2)	0.132
**Cause of CKD**						
Diabetic nephropathy, n (%)	163 (39)	100 (45)	12 (21)	20 (33)	31 (38)	0.011*
Nephrosclerosis, n (%)	74 (18)	46 (21)	10 (18)	13 (22)	5 (6)	0.025*
Glomerulonephritis, n (%)	77 (18)	19 (8)	23 (41)	14 (23)	21 (26)	< 0.001**
Others, n (%)	44 (10)	17 (8)	8 (5)	7 (12)	12 (15)	0.060
Unknown, n (%)	63 (15)	42 (19)	3 (5)	6 (10)	12 (15)	0.183
On THRT, n (%)	45 (11)	18 (8)	8 (14)	7 (12)	12 (15)	0.083
Subclinical hypothyroidism, n (%)	68 (16)	31 (14)	7 (13)	11 (18)	19 (23)	0.044*
Overt hypothyroidism, n (%)	51 (12)	17 (8)	9 (16)	8 (13)	17 (21)	0.002**
Total hypothyroidism, n (%)	119 (28)	48 (21)	16 (29)	19 (32)	36 (44)	< 0.001**
Euthyroidism, n (%)	302 (72)	176 (79)	40 (71)	41 (68)	45 (56)	< 0.001**

*BMI* body mass index, *TSH* thyroid-stimulating hormone, *FT4* free thyroxine, *FT3* free triiodothyronine, *s-Cr* serum creatinine, *eGFRcre* estimated glomerular filtration rate calculated by serum creatinine, *eGFRcys* estimated glomerular filtration rate calculated by serum cystatin C, *24hrCcr* 24-h creatinine clearance, *HbA1c* glycated hemoglobin, *ACE-i* angiotensin-converting-enzyme inhibitor, *ARB* angiotensin II receptor blocker, *DPP-4* dipeptidyl peptidase-4, *GLP-1* glucagon-like peptide-1, *SGLT-2* sodium-glucose cotransporter 2, *SU* sulfonylurea, *TZD* thiazolidinedione, *THRT* thyroid hormone replacement therapy.

*P* for trend was obtained by Cochran-Armitage trend test or Cuzick test. *P < 0.05, **P < 0.01. Data was expressed as n (%) for categorical variables and mean ± standard deviation for continuous variable.

**Table 4:** Nominal logistic regression model for the presence of total hypothyroidism and urinary protein excretion.

Urinary protein	OR [95% CI]	
	Univariate	Multivariate
< 0.5 g/day	0.48 [0.31–0.75]	Reference
0.5–1.49 g/day	1.02 [0.55–1.90]	1.36 [0.66–2.82]
1.5–3.49 g/day	1.21 [0.67–2.18]	0.99 [0.50–1.98]
≧3.5 g/day	2.48 [1.50–4.10]	1.95 [1.06–3.59]

Multivariate: Adjusted for age, sex, 24-h creatinine clearance, body mass index, the presence of hypertension and glycated hemoglobin (HbA1c). OR, odds ratio; 95% CI, 95% confidence intervals.

## Discussion

To the best of my knowledge, this is the first study to investigate the association between 24-h urine protein and hypothyroidism in CKD population. Our study indicated that kidney function, nephrotic range proteinuria, and UP, but not non-nephrotic proteinuria, are significantly correlated with hypothyroidism. In addition, frequency distribution analysis stratified by CKD stage and UP for hypothyroidism clarified the possibility of hypothyroidism in CKD population. It may help for the decision of routine analysis of thyroid function in CKD patients.

There was a significantly increased proportion of hypothyroidism with the progression of CKD, that is in agreement with previous reports^9,31,32^. The levels of FT3, not FT4 or TSH, were correlated with kidney function and UP. It is important because FT3 is the most metabolically active hormone. T3 and thyroxine (T4) are available in circulation as free hormones, which are biologically active^33^. Thyroid hormone molecules can also be activated by conversion from T4 to T3 ^34^. Various factors related to CKD may affect these processes. Considering the higher prevalence of hypothyroidism especially in CKD stage 5, uremic toxin and/or metabolic acidosis due to end-stage renal disease, may be the important cause of hypothyroidism as previously reported^17–19^.

In the point of the association between UP and hypothyroidism, it has been reported that a semi-quantitative measure of proteinuria by urine dipstick is not associated with hypothyroidism^4^. More recently, it is reported that UP is an independent factor associated with hypothyroidism in CKD population^14^. However, most of the participants in the study were end-stage renal disease (46% were CKD stage 5), thus it may not represent the whole CKD population. In addition, urinary protein / creatinine ratio was used in the study. Since it is reported that thyroid function may affect urine creatinine excretion^23^ and urine creatinine excretion decreases as kidney function declines^35^, urinary protein / creatinine ratio might be unreliable in the analysis for the thyroid disorder in CKD population. In this point, our study has the strong point since 24-h urine protein excretion was applied. In addition, participants enrolled in the study are widely distributed into CKD stage 1–5, which may represent the broad CKD population.

The participants with NS showed significantly lower FT4 and FT3 levels as well as higher TSH levels, compared to the participants without NS, indicating that heavy proteinuria, but not mild-moderate proteinuria, may be associated with hypothyroidism. It is reported that proteinuria may cause urinary loss of thyroid hormones bound to the various binding proteins such as thyroxine-binding globulin (TBG), albumin, prealbumin, and transthyretin^20^, which result in the reduction of the serum thyroid hormone levels^21^. Nephrotic range proteinuria, but not non-nephrotic range proteinuria, can be a risk factor for hypothyroidism because the heavier proteinuria may lead more loss of the serum thyroid hormone levels. Our analysis also clarified that nephrotic range proteinuria with DM may not be a risk factor for hypothyroidism, suggesting that the effect of proteinuria on thyroid function depends on the etiologies of CKD. In addition to the effect of heavy proteinuria on hypothyroidism, some causes of NS, such as amyloidosis, systemic lupus erythematosus and mixed connective tissue disease, may lead to hypothyroidism as their possible complications^36–39^. Therefore, hypothyroidism under NS status needs to be differentiated carefully.

Since it has been reported that THRT improved kidney function and slower the decline in kidney function in CKD patients with hypothyroidism^40–43^, the appropriate diagnosis of hypothyroidism, followed by the appropriate treatment may delay CKD progression. Our study highlighted the importance of the routine evaluation of thyroid function in CKD patients, especially for the patients with CKD stage 3–5 and/or with nephrotic range of proteinuria.

There are several limitations in this study. First, the sample size is relatively small in a single university hospital and the participants are mainly hospitalized for education or examination, thus there may be a selection bias. Therefore, it may not represent the CKD population. Nevertheless, considering the patient population enrolled (the highest population is CKD stage 3), it may represent the whole CKD population. Second, this is a retrospective and cross-sectional study, thus the causal relationship between hypothyroidism and CKD cannot be established. Further prospective study is required to clarify the causal relationship. Lastly, thyroid autoantibody or other autoimmune antibodies were not evaluated in this study. Therefore, the possibility of a common underlying autoimmune process related to hypothyroidism cannot be excluded. Nevertheless, it is reported that CKD patients with overt hypothyroidism had 37.5% positive in anti-thyroglobulin antibody (Tg-Ab) and 40% in anti-thyroid peroxidase antibody (TPO-Ab)^14^. Considering high positivity of these antibodies in CKD patients with hypothyroidism, it might be reasonable to include patients with these autoantibodies in the present study.

In conclusion, we re-emphasized the important association between hypothyroidism and CKD. Our study revealed that decreased kidney function and nephrotic range proteinuria, not mild-to-moderate levels of proteinuria, are the independent risk factor of hypothyroidism. Appropriate analysis of thyroid function and appropriate treatment for hypothyroidism in CKD patients are recommended to slow the progression of CKD, and at least a routine analysis of thyroid function in CKD patients with decreased kidney function (CKD stage 3, 4 and 5) or nephrotic range proteinuria would be recommended.

## Supplementary Information

Supplementary Information.

## Data Availability

The datasets used during the current study are available from the corresponding author upon reasonable request.
