# Supplementary material for: The association between hypothyroidism and proteinuria in patients with chronic kidney disease: a cross-sectional study
Source: Sci Rep. 2022 Sep 2;12:14999. doi: 10.1038/s41598-022-19226-0 (PMC9440240; doi:10.1038/s41598-022-19226-0)
Supplement: Supplementary file 1 — Supplementary Information. [file 41598_2022_19226_MOESM1_ESM.pdf]

Supplementary Information

The association between hypothyroidism and proteinuria in patients with chronic kidney disease: A cross-sectional study

Natsumi Matsuoka-Uchiyama, Kenji Tsuji*, Yizhen Sang, Kensaku Takahashi,
Kazuhiko Fukushima, Hidemi Takeuchi, Kenichi Inagaki,
Haruhito A. Uchida, Shinji Kitamura, Hitoshi Sugiyama, Jun Wada

Table S1. Etiologies for CKD of the study participants stratified by CKD stages

Clinical parameters	All CKD (n=421)	Stage 1 (n=34)	Stage 2 (n=92)	Stage 3 (n=177)	Stage 4 (n=65)	Stage 5 (n=53)
Diabetic nephropathy, n	163	19	41	65	24	14
Nephrosclerosis, n	74	2	6	29	19	18
Glomerulonephritis, n	77	7	28	30	4	8
IgA nephritis, n	36	3	13	15	0	5
MN, n	10	0	5	5	0	0
ANCA related, n	7	0	1	3	2	1
FSGS, n	5	1	2	1	0	1
MPGN, n	5	0	1	4	0	0
IgA vasculitis, n	4	2	2	0	0	0
non-IgA CGN, n	1	0	1	0	0	0
PSAGN, n	1	0	0	0	1	0
Lupus nephritis, n	8	1	3	2	1	1
Others, n	44	1	8	17	9	9
TIN, n	11	0	1	7	2	1
TBM, n	2	0	1	1	0	0
MGA, n	2	0	2	0	0	0
MCD, n	6	1	0	3	2	0
ORG, n	3	0	1	0	1	1
Hepatic glomerulosclerosis, n	2	0	0	1	0	1
Amyloidosis, n	4	0	2	1	1	0
Lowe syndrome, n	1	0	0	0	0	1
Pseudo Bartter syndrome, n	2	0	0	1	1	0
TAFRO syndrome, n	1	0	1	0	0	0
Renal infarction, n	1	0	0	1	0	0
PKD, n	4	0	0	0	1	3
Unilateral nephrectomy, n	5	0	0	2	1	2
Unknown, n	63	5	9	36	9	4

MN, membranous nephropathy; ANCA, anti-neutrophil cytoplasmic antibody; FSGS, focal segmental glomerulonephritis; MPGN, membranoproliferative glomerulonephritis; CGN, chronic glomerulonephritis; PSAGN, post-streptococcal acute glomerulonephritis; TIN, tubulointerstitial nephritis; TBM, thin basement membrane; MGA, minor glomerular abnormality; MCD, minimal change disease; ORG, obesity related glomerulopathy; PKD, polycystic kidney disease.

Table S2. Nominal logistic regression models for the presence of total hypothyroidism and renal function.

	OR [95% CI]		
	eGFRcre	eGFRcys	24hrCcr
Model 0	1.32 [1.21-1.45]	1.36 [1.23-1.50]	1.30 [1.20-1.41]
Model 1	1.36 [1.23-1.50]	1.34 [1.22-1.48]	1.31 [1.20-1.43]
Model 2	1.36 [1.22-1.50]	1.34 [1.21-1.47]	1.31 [1.20-1.42]
Model 3	1.32 [1.19-1.47]	1.30 [1.18-1.44]	1.28 [1.18-1.40]

Model 0: Not adjusted. Model 1: Adjusted for age, sex, and BMI. Model 2: Adjusted for age, sex, BMI, and urinary protein. Model 3: Adjusted for age, sex, BMI, urinary protein, and HbA1c. eGFRcre, estimated glomerular filtration rate calculated by serum creatinine; eGFRcys, estimated glomerular filtration rate calculated by serum cystatin C; 24hrCcr, 24-hour creatinine clearance; OR, odds ratio; 95% CI, 95% confidence intervals; BMI, body mass index; HbA1c, glycated hemoglobin. The OR of eGFRcre and eGFRcys are shown by 10 mL/min/1.73m^2 decrease and the OR of 24hrCcr is shown by 10 mL/min decrease.

Table S3. Nominal logistic regression model for the presence of subclinical or overt hypothyroidism and variables.

Subclinical hypothyroidism<br>Clinical parameters	OR [95% CI]<br>Univariate	Multivariate Model 1	Multivariate Model 2
Sex (male)	1.07 [0.63-1.83]	0.80 [0.45-1.40]	0.84 [0.48-1.47]
24hrCcr [decreased by 10 mL/min]	1.20 [1.10-1.32]	1.19 [1.08-1.32]	1.20 [1.08-1.32]
Age	1.01 [0.99-1.03]	1.00 [0.98-1.02]	1.00 [0.97-1.02]
BMI	1.04 [0.99-1.04]	-	-
Hypertension	1.51 [0.84-2.70]	-	-
Urinary protein [increased by 1 g/day]	1.10 [1.04-1.18]	1.09 [1.02-1.16]	-
Nephrotic syndrome	3.25 [1.61-6.66]	-	2.79 [1.33-5.84]
HbA1c	0.32 [0.07-1.51]	-	-
Diabetes mellitus	1.03 [0.61-1.74]	1.08 [0.62-1.88]	1.05 [0.60-1.83]

Overt hypothyroidism<br>Clinical parameters	OR [95% CI]<br>Univariate	Multivariate Model 1	Multivariate Model 2
Sex (male)	0.71 [0.39-1.29]	0.51 [0.26-0.99]	0.52 [0.27-1.00]
24hrCcr [decreased by 10 mL/min]	1.52 [1.32-1.75]	1.47 [1.27-1.69]	1.50 [1.30-1.74]
Age	1.02 [1.00-1.05]	1.01 [0.99-1.03]	1.00 [0.98-1.03]
BMI	0.95 [0.88-1.02]	-	-
Hypertension	2.77 [1.50-5.12]	-	-
Urinary protein [increased by 1 g/day]	1.12 [1.04-1.20]	1.09 [1.01-1.19]	-
Nephrotic syndrome	2.48 [1.08-5.70]	-	2.22 [0.88-5.56]
HbA1c	0.60 [0.46-0.80]	-	-
Diabetes mellitus	0.51 [0.27-0.96]	0.50 [0.25-1.01]	0.52 [0.26-1.04]

OR, odds ratio; 95% CI, 95% confidence intervals; BMI, body mass index; HbA1c, glycated hemoglobin; 24hrCcr, 24-hour creatinine clearance. Multivariate Model1: Adjusted for sex, 24hrCcr, age, urinary protein and the presence of diabetes mellitus. Multivariate Model2: Adjusted for sex, 24hrCcr, age, and the presence of nephrotic syndrome and diabetes mellitus.

Table S4. Characteristics of the study participants in DM and non-DM groups.

Clinical parameters	All CKD (n=421)	DM (n=200)	non-DM (n=221)	P value
Sex (Male), n (%)	231 (55)	114 (57)	117 (53)	0.326
Age (yr)	61±15	63±13	59±17	0.012*
BMI (kg/m^2)	24.6±4.9	25.3±5.0	24.0±4.7	0.001**
TSH (μU/mL)	5.6±18.1	4.9±17.4	6.2±18.7	0.962
FT4 (ng/dL)	1.18±0.22	1.18±0.20	1.18±0.24	0.993
FT3 (pg/mL)	2.48±0.62	2.48±0.50	2.47±0.72	0.668
s-Cr (mg/dL)	1.62±1.34	1.50±1.21	1.74±1.45	0.009**
eGFRcre (mL/min/1.73m^2)	49.6±30.0	52.9±32.4	46.6±27.4	0.024*
eGFRcys (mL/min/1.73m^2)	49.5±30.4	52.0±30.6	47.3±30.0	0.043*
24hrCcr (mL/min)	57.5±36.8	61.2±39.9	54.2±33.5	0.073
Urinary protein (g/day)	2.09±3.72	2.08±3.98	2.10±3.48	0.005**
Albumin (g/dL)	3.5±0.8	3.6±0.7	3.5±0.9	0.157
Hemoglobin (g/dL)	12.1±2.4	12.3±2.4	12.0±2.4	0.049*
Total cholesterol (mg/dL)	194±62	192±57	196±66	0.412
HbA1c (%)	6.8±1.9	8.1±2.0	5.7±0.6	<0.001**
Nephrotic syndrome, n (%)	48 (11)	26 (13)	22 (10)	0.247
Hypertension, n (%)	113 (27)	34 (17)	79 (36)	<0.001**
ACE-i/ARB intake, n (%)	105 (25)	56 (28)	49 (22)	0.226
Cause of CKD				
Diabetic nephropathy, n (%)	163 (39)	163 (82)	0 (0)	<0.001**
Nephrosclerosis, n (%)	74 (18)	12 (6)	62 (28)	<0.001**
Glomerulonephritis, n (%)	77 (18)	13 (7)	64 (29)	<0.001**
Others, n (%)	44 (10)	4 (2)	40 (18)	<0.001**
Unknown, n (%)	63 (15)	8 (4)	55 (25)	<0.001**
On THRT, n (%)	45 (11)	16 (8)	29 (13)	0.065
Subclinical hypothyroidism, n (%)	68 (16)	34 (17)	34 (15)	0.690
Overt hypothyroidism, n (%)	51 (12)	17 (9)	34 (15)	0.009**
Total hypothyroidism, n (%)	119 (28)	51 (26)	68 (31)	0.122
Euthyroidism, n (%)	302 (72)	149 (75)	153 (69)	<0.001**

DM, diabetes mellitus; CKD, chronic kidney disease; BMI, body mass index; TSH, thyroid-stimulating hormone; FT4, free thyroxine; FT3, free triiodothyronine; s-Cr, serum creatinine; eGFRcre, estimated glomerular filtration rate calculated by serum creatinine; eGFRcys, estimated glomerular filtration rate calculated by serum cystatin C; 24hrCcr, 24-hour creatinine clearance; HbA1c, glycated hemoglobin; ACE-i, angiotensin-converting-enzyme inhibitor; ARB, angiotensin II receptor blocker; DPP-4, dipeptidyl peptidase-4; GLP-1, glucagon-like peptide-a; SGLT-2, sodium-glucose cotransporter 2; SU, sulfonylurea; TZD, thiazolidinedione; THRT, thyroid hormone replacement therapy. Data was expressed as n (%) for categorical variables and mean ± standard deviation for continuous variable. P for trend was obtained by Mann–Whitney U test or Pearson's chi-square test. *P <0.05, **P<0.01.

Table S5. Nominal logistic regression model for the presence of total hypothyroidism and variables in DM and non-DM groups.

DM group<br>Clinical parameters	OR [95% CI]<br>Univariate	Multivariate Model 1	Multivariate Model 2
Sex (male)	0.89 [0.47-1.69]	0.68 [0.33-1.41]	0.69 [0.33-1.42]
24hrCcr [decreased by 10 mL/min]	1.35 [1.19-1.54]	1.33 [1.16-1.53]	1.34 [1.17-1.53]
Age	1.02 [0.99-1.04]	1.00 [0.97-1.03]	1.00 [0.97-1.02]
BMI	1.04 [0.98-1.10]	-	-
Hypertension	2.44 [1.12-5.30]	1.42 [0.60-3.39]	1.45 [0.61-3.45]
Urinary protein [increased by 1 g/day]	1.09 [1.01-1.18]	1.03 [0.95-1.12]	-
Nephrotic syndrome	2.46 [1.05-5.77]	-	1.43 [0.57-3.62]
HbA1c	0.75 [0.63-0.92]	-	-

Non-DM group<br>Clinical parameters	OR [95% CI]<br>Univariate	Multivariate Model 1	Multivariate Model 2
Sex (male)	0.92 [0.52-1.63]	0.62 [0.33-1.18]	0.66 [0.35-1.24]
24hrCcr [decreased by 10 mL/min]	1.26 [1.13-1.40]	1.27 [1.13-1.43]	1.28 [1.13-1.44]
Age	1.02 [1.00-1.04]	1.00 [0.98-1.03]	1.00 [0.98-1.02]
BMI	0.99 [0.93-1.05]	-	-
Hypertension	1.68 [0.93-3.01]	0.90 [0.46-1.78]	1.12 [0.59-2.14]
Urinary protein [increased by 1 g/day]	1.16 [1.06-1.27]	1.16 [1.06-1.28]	-
Nephrotic syndrome	3.78 [1.53-9.35]	-	4.53 [1.69-12.17]
HbA1c	0.58 [0.33-1.00]		

DM, diabetes mellitus; OR, odds ratio; 95% CI, 95% confidence intervals; BMI, body mass index; HbA1c, glycated hemoglobin; 24hrCcr, 24-hour creatinine clearance. Multivariate Model1: Adjusted for sex, 24hrCcr, age, the presence of hypertension and urinary protein. Multivariate Model2: Adjusted for sex, 24hrCcr, age, the presence of hypertension and nephrotic syndrome.

Table S6. Nominal logistic regression model for the presence of subclinical or overt hypothyroidism and urinary protein excretion.

Subclinical hypothyroidism	OR [95% CI]	
Urinary protein	Univariate	Multivariate
<0.5 g/day	0.60 [0.35-1.02]	Reference
0.5-1.49 g/day	0.75 [0.32-1.76]	1.08 [0.43-2.71]
1.5-3.49 g/day	1.23 [0.60-2.54]	1.11 [0.50-2.46]
≧3.5 g/day	2.21 [1.19-4.10]	1.84 [0.92-3.68]

Overt hypothyroidism	OR [95% CI]	
Urinary protein	Univariate	Multivariate
<0.5 g/day	0.36 [0.19-0.67]	Reference
0.5-1.49 g/day	1.40 [0.64-3.10]	2.25 [0.84-6.06]
1.5-3.49 g/day	1.18 [0.52-2.70]	1.16 [0.44-3.09]
≧3.5 g/day	2.86 [1.47-5.54]	2.39 [1.02-5.58]

Multivariate: Adjusted for age, sex, and 24-hour creatinine clearance. OR, odds ratio; 95% CI, 95% confidence intervals.

Table S7. Nominal logistic regression model for the presence of total hypothyroidism and urinary protein excretion in DM and non-DM group.

DM group	OR [95% CI]	
Urinary protein	univariate	multivariate
<0.5 g/day	0.39 [0.20-0.74]	Reference
0.5-1.49 g/day	3.79 [1.44-9.96]	3.16 [0.97-10.28]
1.5-3.49 g/day	0.52 [0.17-1.59]	0.41 [0.12-1.44]
≧3.5 g/day	2.50 [1.20-5.25]	1.42 [0.56-3.62]

Non-DM group	OR [95% CI]	
Urinary protein	univariate	multivariate
<0.5 g/day	0.60 [0.34-1.07]	Reference
0.5-1.49 g/day	0.38 [0.15-0.96]	0.80 [0.29-2.24]
1.5-3.49 g/day	1.88 [0.90-3.95]	1.85 [0.79-4.31]
≧3.5 g/day	2.48 [1.24-4.95]	2.87 [1.31-6.30]

Multivariate: Adjusted for age, sex, and 24-hour creatinine clearance. OR, odds ratio; 95% CI, 95% confidence intervals; DM, diabetes mellitus.

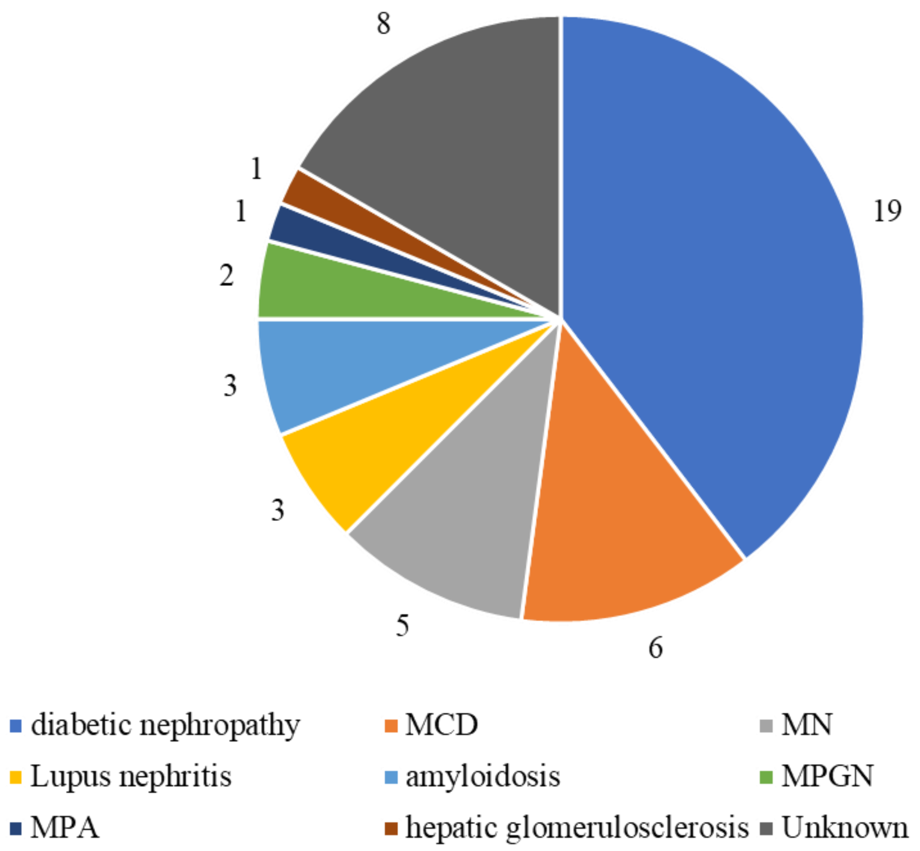

Figure S1. Pile chart of etiologies for CKD in patients with nephrotic syndrome.

MCD, minimal change disease; MN, membranous nephropathy; MPGN, membranoproliferative glomerulonephritis; MPA, microscopic polyangiitis.
